# The time window for the reversal of depigmentation from aggravation to recovery in a non-small-cell lung cancer patient with pre-existing vitiligo using anti-programmed cell death-1 therapy: A case report

**DOI:** 10.3389/fimmu.2022.946829

**Published:** 2022-08-16

**Authors:** Zhiru Gao, Yinghui Xu, Jianjiao Zu, Xu Wang, Chao Sun, Shi Qiu, Ye Guo, Kewei Ma

**Affiliations:** ^1^ Cancer Center, The First Hospital of Jilin University, Changchun, China; ^2^ Dermatological Department, The First Hospital of Jilin University, Changchun, China

**Keywords:** adverse event, AE, Camrelizumab, non-small-cell lung cancer, NSCLC, PD-1

## Abstract

Immune checkpoint inhibitors have made remarkable breakthroughs in the treatment of lung cancer, bringing significant survival benefits to the patients. A number of adverse events aggravated by immunotherapy in patients with pre-existing autoimmune diseases have been reported in the past, especially skin toxicity, such as rash, pruritus, erythema, and vitiligo. However, whether the exacerbated autoimmune disease is reversible and when it will return to its original state after immunotherapy discontinuation is still inconclusive. In our report, we described a patient diagnosed with non-small cell lung cancer whose vitiligo was stable for about 10 years. We followed up and observed the patient’s skin depigmentation for the complete time window, from aggravation of application anti-programmed cell death-1 receptor antibody (anti-PD-1 antibody) to recovery after the withdrawal. We presented the objective images at particular time points using reflectance confocal microscopy and wood’s light. We found that the use of anti-PD-1 antibody aggravated in skin toxicity, but it was reversible, the time window from the beginning to recovery status was approximately 9 months. We used this real case scenario to explain the relationships between immunotherapy and autoimmune diseases.

## Introduction

Immune checkpoint inhibitors (ICIs) primarily work by blocking the signal transduction pathways of the programmed cell death protein-1/programmed cell death ligand 1 (PD-1/PD-L1) or cytotoxic T lymphocyte-associated protein 4 (CTLA-4) (1). These antibodies can principally activate the CD8+ T lymphocyte by preventing the interaction between them and their ligands to generate an antitumor immune response (2). The ICIs significantly improve outcomes in patients with a variety of malignancies, especially for non-small cell lung cancer (NSCLC). However, the ICIs could cause immune-related adverse events (irAEs) through their non-specific positive immunomodulatory effects, and then affect multiple organs of the body. In addition, many previously studies have reported that patients with autoimmune diseases are susceptible to exacerbation of the original autoimmune disease, especially skin toxicity, with rash, pruritus, erythema, and vitiligo being the most common one when receiving immunotherapy (3–5).

Camrelizumab (SHR-1210) is a humanized monoclonal antibody against PD-1. Previous large-scale phase 2 and phase 3 clinical trials have reported that treatment-related skin irAEs of any grade were primarily concentrated in reactive cutaneous capillary endothelial proliferation in cancer patients, with few reports of other skin toxicities (6, 7). Generally, the huge majority of irAEs are reversible if promptly diagnosed and adequately treated (8, 9). Some studies have shown that mild to moderate irAEs (CTCAE Grades 1-2) are largely manageable and reversible within 2 weeks after ICIs discontinuation and treatment based primarily on systemic glucocorticoids, notably methylprednisolone and other immunomodulatory agents (10–12). However, the effect of the ICIs on the underlying condition and subsequent outcome in cancer patients with autoimmune diseases are still indetermined.

Vitiligo is an autoimmune skin disorder, that is defined as hypopigmentation of the skin, which originates from the loss of function of epidermal melanocytes (13). We previously described a diagnosed NSCLC patient with focal vitiligo and her skin depigmentation aggravated in just half a year after the application of anti-PD-1 antibody. The case report was published in the journal of Immunotherapy (14). This time, we reported a gradual recovery status of vitiligo after discontinuation of ICIs, and provided a specific time reference, which should be a further supplementary explanation for follow-up after anti-PD-1 antibody withdrawal. We attempt to illustrate the relationships between the ICIs and autoimmune diseases from the patient’s complete treatment process.

## Case description

### Phase 1: Vitiligo aggravated with the application of anti-PD-1 antibody

**Figure 1:**
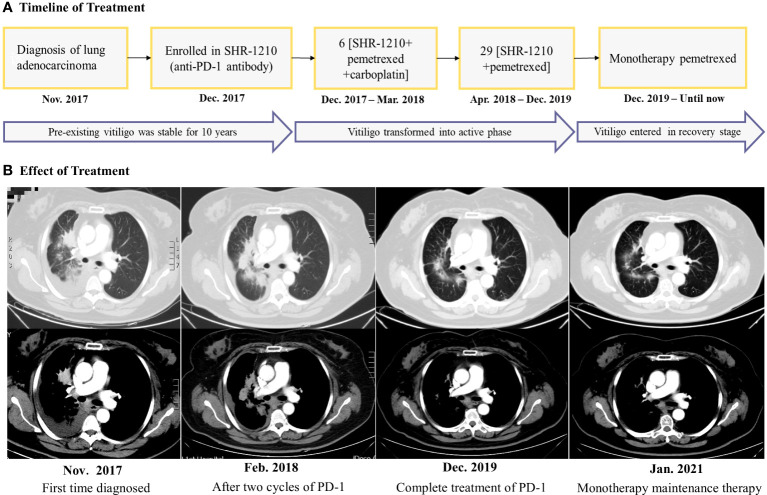
**(A)** Timeline for the diagnosis, treatment, and changes of the vitiligo condition. **(B)** Chest computed tomography scans revealed the clinical response during the treatment. The efficacy evaluation was stable disease after the two cycles of ‘SHR-1210 combined with pemetrexed and carboplatin, but as the treatment time was prolonged, the tumor density gradually decreased, suggesting that the tumor has been effectively controlled.

A 62-year-old female came to our hospital due to cough and chest tightness. She underwent chest computed tomography examination and found space-occupying lesions and large pleural effusion in the right lung in November 2017(**Figure 1B**). After pathological and systemic examination, the patient was diagnosed with stage IV lung adenocarcinoma with negative gene mutations (EGFR/ALK/ROS1). In addition, the patient had a history of vitiligo for 10 years. The vitiligo lesions were confined to the area around the eyes and mouth of the face and remained stable without any treatment. To further treat lung cancer, she participated in phase III, randomized, open-label, multicenter study of SHR-1210 (anti-PD-1 antibody) combined with pemetrexed and carboplatin as the first-line treatment for patients with the advanced or metastatic non-squamous NSCLC. She was randomly assigned to an experimental group in December 2017 and received 6 cycles of “SHR-1210 combined with pemetrexed and carboplatin” regimens, followed by 29 cycles of “SHR-1210 in combination with pemetrexed” as subsequent therapy (**Figure 1A**). After the first two cycles of treatment in February 2018 (6 weeks), lung cancer was effectively controlled, and the efficacy evaluation was stable disease(**Figure 1B**). Moreover, she suffered from another major irAEs, hypothyroidism (CTCAE 4.0 one grade). And 75μg euthyrox (levothyroxine) was administered once a day from February 28, 2018, to control the immune-related hypothyroidism.

**Figure 2:**
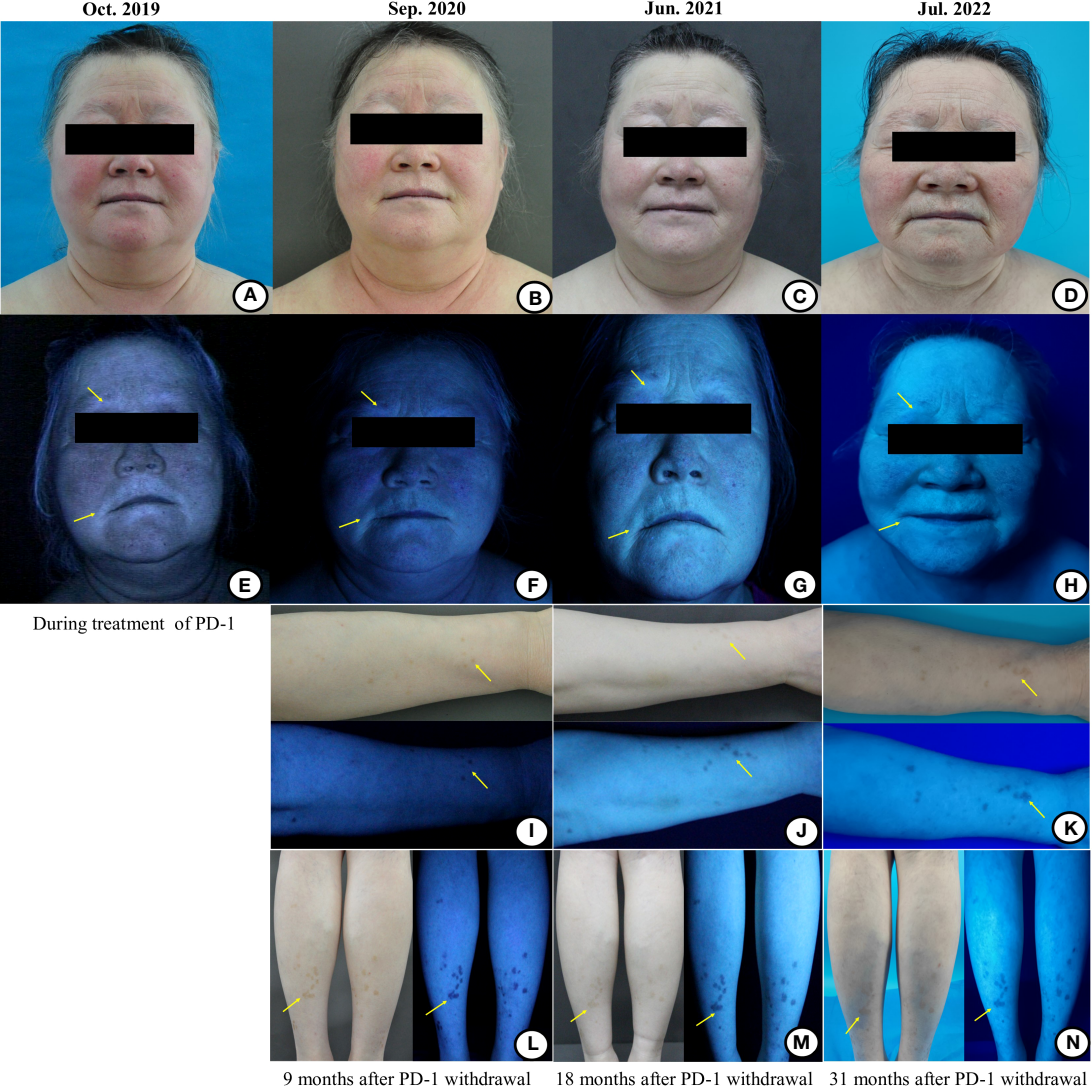
The images of the patient’s facial and limbs changes under the natural light and wood’s light during and after the treatment of the anti-PD-1 antibody. **(A)** During the treatment of immunotherapy, the vitiligo areas on the face were gradually enlarged, even involving the hair (including hair on the head/eyelashes/eyebrows), which showed depigmentation. **(B–D)** With the prolongation of immunotherapy withdrawal, the vitiligo areas on the face were gradually narrowed and the hair was gradually turned black. **(E)** Wood’s light examination showed that the vitiligo areas were bright bluish-white, and the hypopigmentation area was also found at the junction of the normal and depigmented skin. The hypopigmentation areas around the mouth under wood’s light examination presented a bright bluish-white signal. **(F–H)** With the prolongation of the immunotherapy withdrawal, the bright bluish-white areas on the mouth-around were gradually narrowed and faded. **(I, L)** Scattered pigmented spots began to emerge on the patient’s right forearm and lower legs under the wood’s light examination 9 months after stopping the immunotherapy. **(J, M)** The pigment macules of the right forearm and the lower legs became lighter than before under the natural light 18 months after stopping the immunotherapy, which may be related to the lack of sunlight during the winter in northern China. Under the wood’s light, the amount and range of pigmentation of the right forearm were increased than before, while the macules on lower legs were stable 18 months after the immunotherapy withdrawal. **(K, N)** With the prolongation of the immunotherapy withdrawal, the amount and range of pigmentation of the right forearm and lower legs were further increased and expanded than before.

**Figure 3:**
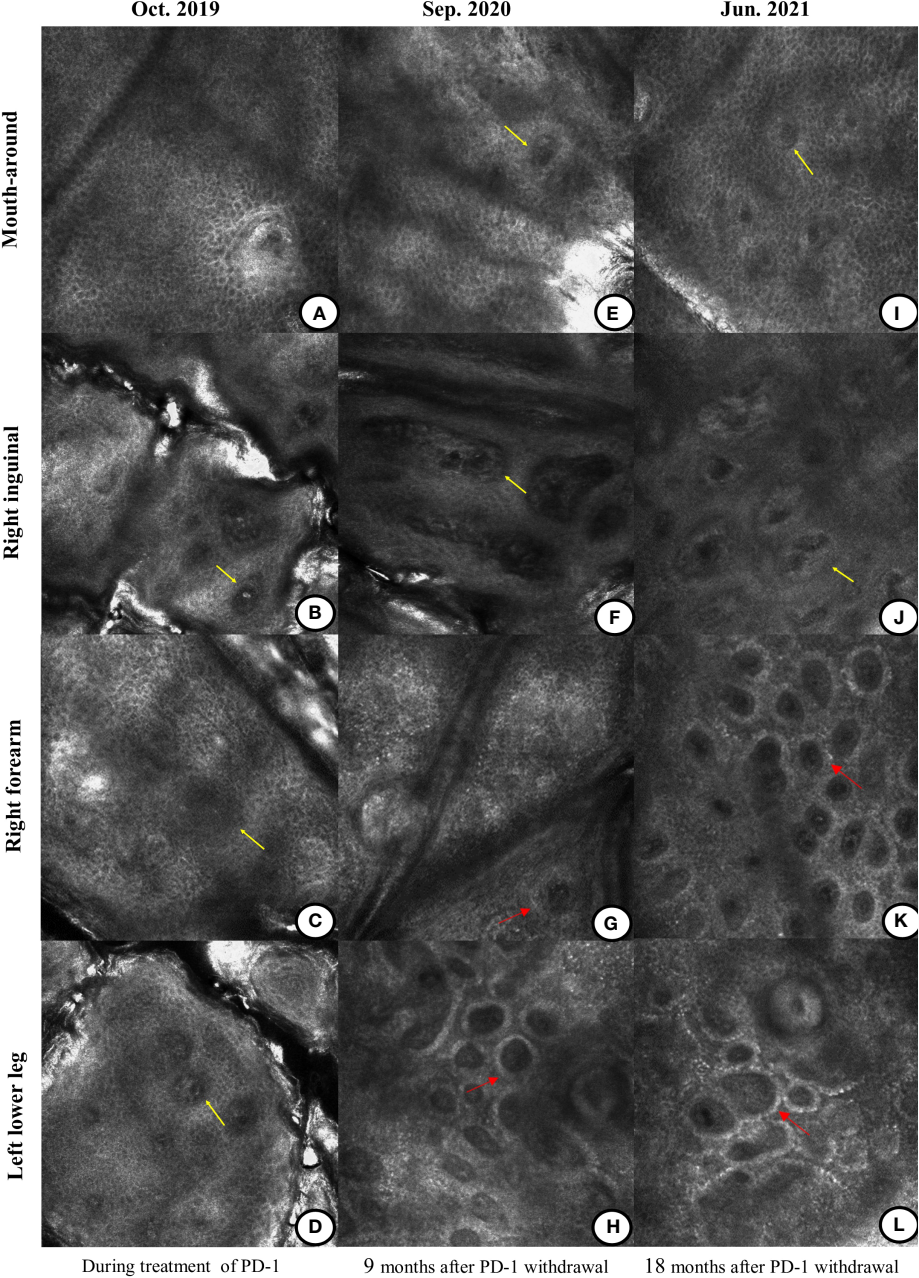
Differences in the reflectance confocal microscopy (RCM) images during and after the treatment of the anti-PD-1 antibody. **(A–D)** During the treatment of immunotherapy, the RCM images displayed the absence of pigment rings in the basal cell layer of the mouth-around, right inguinal region, right forearm, and left lower leg (yellow arrow). **(E–H)** Nine months after immunotherapy was discontinued, there was still no pigment ring on the mouth-around and right inguinal region (yellow arrow). Pigment rings gradually appeared on the right forearm and the left lower leg (red arrow). **(I–L)** Eighteen months after the immunotherapy withdrawal, there were no pigment rings in the mouth-around and right inguinal region (yellow arrow). But on the right forearm and left lower leg, the pigment rings became brighter (red arrow).

During immunotherapy, we found that the vitiligo in the patient rapidly aggravated with depigmentation of the skin over the whole body in just half a year. Her facial depigmentation area began to expand around the mouth and eyes, and the depigmentation lesions gradually appeared in other parts of the body, such as the inguinal region, cheeks, limbs, etc. Additionally, her hair (on the head, eyelashes, and eyebrows) also gradually turned white (**Figures 2A, E**). No corticosteroids or other treatments were administered during this period. Written informed consent was obtained from the patient for the use of the images. Reflectance confocal microscopy (RCM) images were captured from different parts of the patient’s body. The RCM images of the patient showed almost the absence of pigmented cells and pigmented rings on the whole skin(**Figures 3A–D**). At the stage of the application of the anti-PD-1 antibody, we observed that the patient’s skin depigmentation began to aggravate in just half a year. We recorded the imaging pictures with the most aggravated condition in October 2019 (about 22 months of using anti-PD-1 antibody) and have published them as a form of the case report (14).

### Phase 2: Vitiligo recovery after discontinuation of the anti-PD-1 antibody

According to the provisions of this clinical study, the immunotherapy was discontinued after 2 years (total of 35 cycles) in December 2019 and single agent pemetrexed was started as maintenance therapy until now (**Figure 1A**). However, since the discontinuation of the anti-PD-1 antibody, we were surprised to find that the patient’s vitiligo condition gradually recovered. In the beginning, we found that depigmented areas of the eyes and mouth-around were slightly narrowed and the hair gradually began to darken (**Figures 2B, F**). Furthermore, we found that the patient began to have marked pigmentation on the forearms and lower legs (**Figures 2I, L**). The RCM images of the patient also showed that the pigment rings were gradually appeared in the forearms and left lower legs but there were no significant changes on the mouth-around and inguinal region(**Figures 3E–H**). After 9 months of discontinuation of immunotherapy, we observed that the skin depigmentation of patients began to recover gradually, and we found that the time to start recovery was longer (about 9 months after discontinuation of anti-PD-1 antibody) than the time to aggravate (about 6 months after using the anti-PD-1 antibody) the vitiligo.

About 18 months after the immunotherapy withdrawal (June 2021), we found that the patient’s pigment recovery was more apparent. The specific manifestation was that the patient’s hair was darker than half a year before, and the facial depigmentation areas were also gradually narrowed and faded(**Figures 2C, G**). Although the pigmentation of the forearms and lower legs became lighter under the natural light, we speculated that it may be related to the lack of sunlight during the winter in northern China. Moreover, the number and extent of pigmentation of the right forearm have increased than before, while the macules on the lower legs were stable under the wood’s light (**Figures 2J, M**). The RCM images showed that there was still no pigment right around the mouth-around and inguinal region. Moreover, there were more and brighter pigment rings and pigment cells on the forearms and lower legs than before(**Figures 3I–L**). About 31 months after the immunotherapy withdrawal (July 2022), we found that the vitiligo areas on the face were narrower and lighter, and the hair became darker than before (**Figures 2D, H**). The amount and range of pigmentation of the right forearm and lower legs were also increased and expanded compared to before (**Figures 2K, N**). With the extension of the treatment time, the tumor density gradually decreased, suggesting that the patient reached a durable stabilization of her tumor (**Figure 1B**). At the stage of discontinuation of immunotherapy for more than 2 years, we observed gradual recovery after the immunotherapy withdrawal and noted a trend from distal to proximal. In brief, the toxic effects of PD-1 inhibitors to the skin were reversible.

## Discussion

In recent years, immunotherapy is considered to play a central role in the treatment of cancers, as shown in lung cancer. However, some studies found that the patients with autoimmune diseases are susceptible to the ICIs and are associated with a significantly increased risk of mortality related to the irAEs. About 40% of the patients have different degrees of skin toxicity, and most of the lesions are mild to moderate and can be well controlled by symptomatic supportive treatment (15, 16). The specific mechanism of the irAEs occurrence is still inconclusive, but it is generally believed that the overreactive immune response may be caused by the autoreactive T cells, triggering the related symptoms of the corresponding organs (17). The ICIs that differ from the traditional cytotoxic or molecularly targeted drugs, do not follow a periodic pattern like the traditional chemotherapeutic agents, and the time of toxicity is usually delayed and persistent. The research showed that although the irAEs occur at different times, they usually take place within 1-6 months and are mostly reversible after the drug withdrawal (18).

In this case report, we presented a patient with the stage IV NSCLC with pre-existing vitiligo for about 10 years. The patient was treated with the anti-PD-1 antibody (SHR-1210) for about 2 years and obtained a durable stabilization of her primary tumor during the subsequent maintenance chemotherapy. Our previous report described that the application of the anti-PD-1 antibody accelerated the generalized depigmentation of the skin in just half a year. This time we followed the complete time window after the immunotherapy withdrawal and presented objective images at particular points using reflectance confocal microscopy and wood’s light. After the discontinuation of immunotherapy, the patient’s vitiligo began to gradually enter the recovery phase. For about 9 months after stopping the immunotherapy, we found that the range of skin depigmentation was gradually reduced, the hair was gradually darkened, and there was pigmentation at the forearms, lower legs, and other distal ends. We observed these changes in general and found that the skin changes caused by the anti-PD-1 antibody were reversible. Because the condition of the skin was deteriorated after the treatment with the anti-PD-1 antibody and recovered after the drug withdrawal during the relatively complete-time window. We also found that the patient’s skin depigmentation was gradually recovered without any hormone treatment and the recovery time was longer than the occurrence time. Therefore, the aggravation or regression of skin changes does not influence the curative effect and has no substantial correlation with the clinical curative effect.

Vitiligo is a defective skin disorder, characterized by depigmentation due to the destruction of the epidermal melanocytes, accounting for 0.5%-1% in the general population, which pathogenesis is complex and often associated with the genetic and autoimmune conditions (19). The research indicated that dysfunction of T regulatory cells (Tregs) and hyperactivation of CD8+ cytotoxic T cells often occur at the edge of the depigmented skin (20, 21). Moreover, compared with the T cells isolated from typical vitiligo patients or healthy skin, the CD8^+^ T cells in patients with the immune-related vitiligo produced a higher proportion of interferon-γ (IFN-γ) or both IFN-γ and tumor necrosis factor α (TNFα) (22). Therefore, some studies showed that skin depigmentation by the immune-related vitiligo may be associated with the weakening of the Tregs, which leads to hyperactivated T cells attacking normal melanocytes *in vivo* (23, 24). Specific to the treatment, the asymptomatic vitiligo is commonly observed without therapy since it does not directly threaten the lives of the patients. Most of them can maintain immunotherapy while using good sunlight protection. Depigmented spots can be treated with topical glucocorticoids, calcineurin inhibitors, or combined with phototherapy (25). However, the therapeutic effect of the immune-related vitiligo remains inconclusive. One case of nivolumab-induced vitiligo was successfully treated with narrowband ultraviolet light therapy (26). But in another study, pembrolizumab-associated vitiligo-like depigmentation was not improved by the combination of the excimer laser and topical corticosteroid therapy (27). In our study, the patient did not receive any hormones or other treatment for vitiligo since her quality of life was not additionally affected.

Vitiligo-like lesions are one of the typical side effects occurring in patients receiving anti-PD-1 antibodies. A recent study showed that melanoma cell destruction was associated with the release of melanoma-associated antigens. Therefore, the appearance of the vitiligo is an indicator of melanocyte antigen immune activation and an independent favorable prognostic factor, but it has not been confirmed in patients with the NSCLC (28, 29). Previous data intensively showed that the dermatological irAEs during the anti-PD-1 treatment were correlated with the overall survival (OS) and can be used as a parameter to forecast a better response to the treatment (30–32). The criteria for evaluating the vitiligo disease include the Koebner phenomenon, Wood’s light examination, and RCM imaging. Among them, the RCM is the most suitable non-invasive method for detecting skin melanin abnormalities. Under the RCM, the melanocytes and pigmented keratinocytes of the skin can be seen as bright pigment ring structures on a dark background (33). According to the above diagnostic criteria, the patient’s forepassed vitiligo condition was stable for 10 years, and the vitiligo became worse after using the anti-PD-1 antibody for about half a year. The patient’s vitiligo was transformed from stable to progressive, and the vitiligo disease activity (VIDA) score changed from 0 to + 4. One possible explanation is that immunotherapy activates the T cells, which in turn makes the CD8+ cells overactive, thereby rendering autoimmune susceptibility (34). After stopping the anti-PD-1 antibody for about 9 months, the patient developed skin pigmentation and gradually blackened hair, and the vitiligo changed from progressive to stable. Eighteen months after the drug was discontinued, the patient’s vitiligo remained stable for more than one year without further expansion, and the VIDA score changed from + 4 to -1, which showed vitiligo disease gradually recovered. In addition, the RCM images showed that the pigment cells and pigment rings were gradually recovered. We speculated that the cause of the recovery of the vitiligo may be associated with the gradual recovery of the CD8+ T cells after the drug withdrawal.

Before PD-1 treatment, the patient’s vitiligo lesions were located around the mouth, nose, eyes and along the hairline of the forehead, and the vitiligo extent score (VESplus) was supposed to be 0.011 (35, 36). After about 2 years of PD-1 treatment, the depigmented lesions began to spread around her face, the whole body surface except the hair (hair along the forehead hairline was also depigmented) was depigmented, and the VESplus score was 0.997. After stopping the anti-PD-1 antibody, the pigment spots began to emerge on her lower legs, forearms and face gradually until recently (July, 2022). Therefore, according to the density of the re-pigmented spots, the VESplus score was 0.992. As we can’t get access to the online resources of vitiligo calculator, the value of VESplus score was the average score assessed by two dermatologists in our hospital independently.

In conclusion, we reported a NSCLC patient with pre-existing vitiligo and completely followed up the changes of skin pigmentation during and after anti-PD-1 antibody treatment. The observation for the time window of the treatment showed that the skin toxicity caused by the ICIs was reversible, the time window for onset of recovery was 9 months, and the recovery time was longer than the aggravation time. However, this is just one case report, and more cases are needed to verify its reversibility and time-limited recovery. For other life-threatening irAEs, such as interstitial pneumonia or myocarditis, whether the treatment is necessary and how long it will take to restore the functions of the body’s various organs after stopping the ICIs still needs more population samples to verify.

## Data availability statement

The original contributions presented in the study are included in the article/Supplementary Material. Further inquiries can be directed to the corresponding author.

## Ethics statement

The studies involving human participants were reviewed and approved by the institutional review board of The First Hospital of Jilin University. The patients/participants provided their written informed consent to participate in this study. Written informed consent was obtained from the individual(s) for the publication of any potentially identifiable images or data included in this article.

## Author contributions

KM were responsible for study design and concept. ZG and YX performed data analysis and drafted the manuscript. JZ and XW revised the manuscript. SQ and YG reviewed the article critically for important intellectual content. All authors contributed to the article and approved the submitted version.

## Conflict of interest

The authors declare that the research was conducted in the absence of any commercial or financial relationships that could be construed as a potential conflict of interest.

## Publisher’s note

All claims expressed in this article are solely those of the authors and do not necessarily represent those of their affiliated organizations, or those of the publisher, the editors and the reviewers. Any product that may be evaluated in this article, or claim that may be made by its manufacturer, is not guaranteed or endorsed by the publisher.
